# A Bio-Inspired Approach to Sustainable Building Design Optimization: Multi-Objective Flow Direction Algorithm with One-Hot Encoding

**DOI:** 10.3390/biomimetics11010031

**Published:** 2026-01-02

**Authors:** Ahmet Serhan Canbolat, Emre İsa Albak

**Affiliations:** 1 Department of Mechanical Engineering, Engineering Faculty, Bursa Uludağ University, Bursa 16059, Türkiye; ascanbolat@uludag.edu.tr; 2 Department of Automotive Engineering, Engineering Faculty, Bursa Uludağ University, Bursa 16059, Türkiye

**Keywords:** carbon footprint, external wall insulation, multi-objective optimization, one-hot encoding, Pareto optimality, discrete optimization

## Abstract

The urgent need for sustainable building design calls for advanced optimization methods that simultaneously address economic and environmental objectives, particularly those involving mixed discrete-continuous variables such as insulation material, heating source, and insulation thickness. While nature-inspired metaheuristics have shown promise in engineering optimization, their application to building envelope design remains limited, especially in handling discrete choices efficiently within a multi-objective framework. Inspired by the natural process of rainwater runoff and drainage basin dynamics, this study presents a novel hybrid approach integrating the Multi-Purpose Flow Direction Algorithm (MOFDA) with One-Hot Encoding to optimize external wall insulation. This bio-inspired algorithm mimics how water seeks optimal paths across terrain, enabling effective navigation of complex design spaces with both categorical and continuous variables. The model aims to minimize total lifecycle costs and CO_2_ emissions across Türkiye’s six updated climatic regions. Pareto-optimal solutions are created using MOFDA, after which the Complex Proportional Assessment (COPRAS) method, weighted by Shannon Entropy, selects the most balanced designs. The results reveal significant climate-dependent variations: in the warmest region, the cost-optimal thickness is 3.3 cm (Rock Wool), while the emission-optimal reaches 17.3 cm (Glass Wool). In colder regions, emission-driven scenarios consistently require up to 40 cm insulation, indicating a practical limit of current materials. Under balanced weighting, fuel preferences shift from LPG in milder climates to Fuel Oil in harsher climates. Notably, Shannon Entropy assigned a weight of 88–92% to emissions due to their wider variability across the Pareto front, underscoring the environmental priority in data-driven decisions. This study demonstrates that the bio-inspired MOFDA framework, enhanced with One-Hot Encoding, effectively handles mixed discrete-continuous optimization and provides a robust, climate-aware decision tool for sustainable building design, reinforcing the value of translating natural flow processes into engineering solutions.

## 1. Introduction

The escalating global climate crisis has called for a major energy policy revision. Reducing carbon emissions has become the new primary focus of energy policies, whereas before, it was only a matter of managing the energy supply. One of the important elements of this change is a joint framework, such as the European Green Deal, that aims to convert Europe into the first climate-neutral continent by 2050. In this way, the European Union (EU) moves beyond its borders through a parallel instrument—the Carbon Border Adjustment Mechanism (CBAM), which is helping to slow down climate issues worldwide by making carbon emissions less cost-effective for industries and economies everywhere [1]. The building sector under these circumstances is still the most critical area where the battle will be fought. As the one accountable for around one-third of the global energy demand and a considerable share of greenhouse gases, the built environment is the biggest source of low-carbon solutions at affordable costs [2]. Since the building envelope is the main interface for heat exchange, reducing heat losses through external walls is not only an energy-saving measure but also a fundamental requirement to comply with these international carbon neutrality standards. As a result, thermal insulation enhancement has moved beyond the issue of thermal comfort to become an essential part of sustainable development and emission reduction strategies.

Energy efficiency and environmental impact reduction in buildings can be best achieved through wall exterior insulation. Several methods have been proposed to determine the best insulation parameters in this area. A review of the literature’s evolution reveals that the optimization of building insulation was initially regarded mainly as an economic problem that involves minimizing the investment and operating costs. For instance, Bademlioglu et al. [3] utilized the Taguchi method to analyze the changes in parameters influencing insulation thickness and total costs, and they identified the number of heating degree days as the most critical factor. Equally, Canbolat [4] set a similar economic framework for the problem. He studied the influences of solar radiation and façade orientation on different seasonal usage scenarios, such as summer houses, winter houses, and all-season houses, and determined the cost-oriented optimum insulation thicknesses and payback periods for each case, also including intermediate orientations. Rosti et al. [5] examined the thermal behavior of four different wall constructions, i.e., typical brick walls and modern wall types such as hollow clay, LECA, and AAC blocks in eight cities that represent various climate zones in Iran, for the four main directions, for four seasons. By changing the insulation thickness between 0.5 cm and 9.5 cm, and at the same time, without the help of any complicated optimization algorithms, they carried out the heat transfer loads calculated by numerical methods, and then a Life Cycle Cost (LCC) analysis was applied, to find the optimal insulation thickness, energy savings, and payback period. Moreover, Ozel [6] moved this economic view further by employing a dynamic thermal model to predict how different structural wall materials (e.g., concrete, briquette, and aerated concrete) would affect the thermal performance, and thus came to the conclusion that the optimum insulation thickness and energy savings change substantially with the wall’s heat capacity and conductivity.

Nevertheless, the growing environmental concerns due to climate change have made it necessary to consider not only the financial performance of insulation systems but also their carbon footprint. Axaopoulos et al. [7] demonstrated that a method solely focused on reducing CO_2_ emissions requires a much thicker insulation layer compared to cost-driven strategies, thus highlighting the trade-off between these two objectives. As part of this sustainability-oriented transition, Kitsopoulou et al. [8] researched passive building envelope technologies, suggesting that vacuum insulation panels and aerogel-based materials could achieve energy savings of up to 68.7% in winter and 30.0% in summer in residential buildings; however, they come with difficulties in installation due to their high prices. Investigating the use of environmentally friendly materials, Deshmukh and Yadav [9] explored the potential of composite materials made of bamboo biochar, fly ash, and lime; they proved that bamboo biochar can become a good insulation substitute, having a U-value in the range of $0.51–0.60 W/m^2^K as well as carbon sequestration properties, while fly ash lowers the thermal conductivity of concrete and at the same time increases its durability. To facilitate the decision-making process, Anastaselos et al. [10] created a comprehensive evaluation instrument grounded on the Life Cycle Assessment (LCA) approach; by contrasting the performance of traditional double brick walls with that of External Thermal Insulation Composite Systems (ETICS), they concluded that the application of ETICS might lead to a reduction in energy consumption and CO_2_ emissions by as much as 20% during both the construction and operation stages. Moreover, the same authors, Özkan and Onan [11], used the P1-P2 method to quantify the decreases in CO_2_ and SO_2_ emissions together with the economic costs for various degree-day regions in Türkiye; they analyzed the impact of different factors such as insulation material and fuel type on the results.

Studies in the recent past have broadened the range of optimization models to integrate environmental and economic objectives simultaneously. Within this framework, Canbolat [12] used a multi-objective optimization with the ‘knee-point’ method for the first time to minimize the overall cost and CO_2_ emissions at the same time. He combined fuel parameters (natural gas, electricity, and fuel oil) and insulation material choices to formulate the environmental equations for optimal insulation thickness and discovered that a CO_2_-oriented optimization always gives higher insulation thicknesses than a cost-oriented one. By incorporating the dimensions of the decision-making process, Kilis et al. [13] merged Mathematical Programming and Multi-Criteria Decision-Making (MCDM) methods. In their research, besides type and thickness of insulation material, they also considered the choice of window frame material (aluminum, PVC, and wood) as a design variable. Corresponding to these variables, they optimized simultaneously the conflicting objectives of economic cost, embedded energy (energy footprint), environmental costs (CO_2_ emissions), and thermal resistance maximization. Behzadi Hamooleh et al. [14] focus on a multi-objective optimization problem, where they explored the effect of PCM in the building envelope besides insulation. They conducted a study using Response Surface Methodology (RSM) on six design variables, among which cooling/heating thermostat set points, insulation properties, and PCM characteristics were included. In order to address the problem of discrete design variables with considerable complexity, Canbolat and Albak [15] implemented evolutionary algorithms, NSGA-II, to optimize wall configurations. Their research invited the variable of wall material types (light concrete, brick, reinforced concrete) to the study along with insulation and heating systems, thus enlarging the design space and pictorially explaining the performance of the algorithm in dealing with multidimensional design problems.

Biomimetic, or biologically inspired design, aims to emulate models, systems, and elements from nature to solve complex human problems. In engineering optimization, this approach typically involves adopting algorithms that mimic natural processes such as evolution, swarm behavior, or physical events [16,17,18,19,20]. The Flow Direction Algorithm (FDA), a meta-heuristic optimization method inspired by the process of rainwater transformation into surface runoff in a drainage basin, is one of the recently introduced approaches [21]. Elfatah et al. [22] proposed a modified version of the Flow Direction Algorithm (mFDA) to determine the optimal sizing of a hybrid PV/diesel/battery system in Luxor, Egypt, aiming to minimize the Net Present Cost (NPC), Cost of Energy (COE), and Loss of Power Supply Probability (LPSP) while achieving superior convergence performance compared to other metaheuristic algorithms. Building upon such applications in single-objective optimization, the principles of biomimetic design naturally extend to more complex problem formulations. In addition to hybridization, biomimetic optimization algorithms are frequently used in transformations to multi-objective optimizations. This leads to the development of multi-objective variants, such as the Multi-Objective Flow Direction Algorithm (MOFDA), which adapts the core rainwater runoff-inspired mechanics to efficiently handle and balance competing objectives, like cost, reliability, and performance, within a single optimization framework [23].

This research presents a comprehensive and novel method for optimizing building insulation based on a biomimetic approach. The fundamental innovation lies in the integration of the Multi-Objective Flow Direction Algorithm (MOFDA), a meta-heuristic inspired by nature that mimics the hydrological processes of surface flow and drainage basin dynamics, with the one-hot encoding approach. This biomimetic optimization algorithm, which mimics how water naturally seeks the most suitable paths across terrain, has been used to solve a multi-objective optimization problem aimed at minimizing both life-cycle costs and CO_2_ emissions for building exterior wall insulation. Subsequently, the Complex Proportional Assessment (COPRAS) method is applied to select the most balanced design from the Pareto-optimal set. The weightings required for COPRAS in the best design selection phase were obtained using the Shannon Entropy approach, a concept rooted in thermodynamics and information theory that quantifies disorder and uncertainty in both natural and engineered systems. By merging bio-inspired optimization, intelligent encoding of discrete choices, and nature-informed decision analysis, this work not only offers a significant methodological contribution to building insulation system design but also exemplifies the effective translation of natural principles into sustainable engineering solutions.

## 2. Theoretical Background and Model Structure

### 2.1. Heating Degree-Day Formulation Accounting for Solar Influence

The degree-day method represents a basic tool for calculating one of the most essential energy demands: heating. Even though the conventional technique operates on dry-bulb temperature alone, it is advisable to consider solar radiation effects in this type of analysis if one is keen on having accurate results. In such situations, sol-air temperature (*T_sol_*) is a better representative of the outdoor temperature than the daily average temperature (*T*_0_).

Determining *T_sol_* starts with calculating the incident solar radiation (${\dot{q}}_{s}$), which is made up of direct, diffuse, and reflected components, in addition to the properties of the surface such as solar absorptance and convective coefficients. The comprehensive mathematical model and the derivation of each step for these solar radiation components were very detailed in our previous publication [15]. To escape a long and tedious explanation, we take the same approach as before for the computation of solar parameters in this research.

Consequently, the HDD values accounting for solar influence are calculated using the derived sol-air temperature as follows:

(1) $$HDD=\sum_{d=1}^{365}(T_{b}-T_{0})\;for\;T_{b}>T_{0}$$

where *T_b_* represents the base temperature.

### 2.2. Annual Heat Loss Estimation Through Building Walls

**Figure 1:**
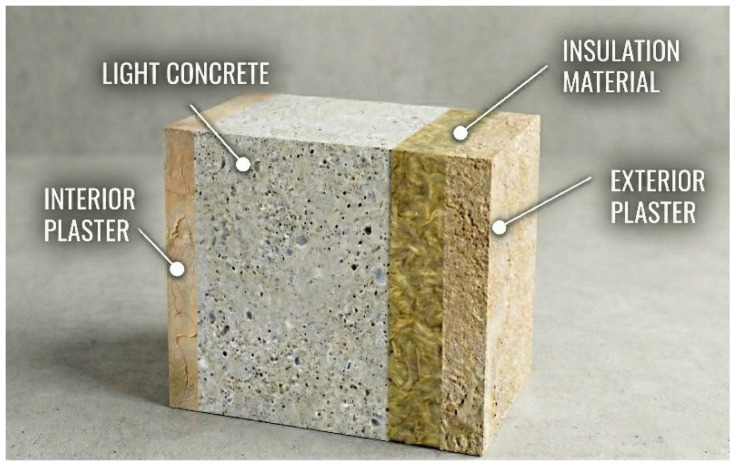
Layered structure of the wall assembly used in the study.

Calculating heat loss through external walls during the heating season is crucial in determining the annual energy requirements of buildings. For this purpose, Heating Degree Days (HDD) values are taken into account within the scope of the study, and heating loads related to the winter period are determined. The wall structure evaluated in this study has a fixed composition consisting of 2 cm interior plaster, 20 cm light concrete, and 3 cm exterior plaster (see Figure 1). This structure is one of the traditional wall sections widely used in residential buildings in Türkiye and is used unchanged for all scenarios in the analyses. In this study, the thermal conductivity of light concrete is assumed to be 0.71 W/mK, based on values reported in the literature for higher-density lightweight concrete commonly used in exterior wall applications, rather than ultra-light insulating concrete (see Table 1).

To calculate the annual heat loss through external walls, it is necessary to determine the thermal resistance of each layer constituting the wall, as well as the film resistances on the inner and outer surfaces. In the study, the internal and external surface heat transfer coefficients are taken as 8.29 W/m^2^K and 28.35 W/m^2^K, respectively [15]. The film resistances corresponding to these values are defined by the classical approach relations $R_{i}=1/h_{i}$ and $R_{o}=1/h_{o}$. The total thermal resistance of the uninsulated part of the wall, $R_{w}$, is calculated based on the thermal conductivity coefficients and thicknesses of the interior and exterior plaster and lightweight concrete layers. Since the insulation material is examined as a variable parameter in this study, the thermal resistance of the insulation layer, $R_{ins}$, is determined according to the thermal conductivity value and thickness of the selected material (see Table 1).

The annual heat loss occurring through external walls during the heating season is calculated using the HDD value with the following expression [15].

(2) $$q_{A,H}=\frac{86,400\;HDD}{\left(R_{i}+R_{o}+R_{w}+R_{ins}\right)}$$

Annual energy consumption is obtained by dividing the heat loss by the efficiency of the heating source used:

(3) $$E_{A,H}\;=q_{A,H}/\eta$$

Here, η represents the efficiency of the selected heating system. Since different fuel types are analyzed in this study, efficiency values are taken from the relevant literature and vary according to the scenarios (see Table 2). The efficiency values (η) assigned to the heating sources represent the practical end-use performance of the heating equipment.

### 2.3. Economic Evaluation and CO_2_ Emission Modeling

In this study, the Life Cycle Cost (LCC) analysis method is adopted to evaluate the cost-effectiveness and environmental benefits of applying thermal insulation to the exterior walls of buildings. This approach aims to minimize the total cost by discounting both the initial investment cost of insulation and the heating energy expenses incurred throughout the building’s service life to their present value. The present value of future energy expenditures is determined using the Present Worth Factor (PWF), which varies according to the inflation rate (g) and the interest rate (i). In this study, the inflation rate is taken as 17% and the interest rate as 20%. These values reflect the officially published indicators of the Turkish Statistical Institute and the Central Bank of Türkiye [24,25]. In this study, the PWF is calculated using Equation (4):

(4) $$PWF=\frac{{\left(\frac{1+i}{1+g}\right)}^{N}-1}{{\left(\frac{i-g}{1+g}\right)\left(\frac{1+i}{1+g}\right)}^{N}}$$

Here, *N* represents the economic lifetime of the investment. Following the insulation application, the annual heating energy cost of the building ($C_{Heat}$) is calculated using Equation (5), which incorporates the Heating Degree Days (HDD), the lower heating value of the selected fuel ($H_{u}$), the system efficiency (η), and the unit price of the fuel ($C_{f}$).

(5) $$C_{Heat}=\frac{86,400HDDC_{f}PWF}{\left(\frac{1}{h_{i}}+\frac{1}{h_{o}}+\frac{x_{plast}}{k_{plast}}+\frac{x_{wall}}{k_{wall}}+\frac{x_{ins}}{k_{ins}}\right)H_{u}\eta }$$

The total annual cost $C_{T,A}$ consists of the insulation material cost, the installation/labor cost $C_{\mathrm{inst}}$, and the lifetime heating energy cost. The overall cost function is presented in Equation (6):

(6) $$C_{T,A}=C_{ins}x_{ins}{\;+\;C}_{inst}+C_{Heat}$$

Five insulation materials, specifically Expanded Polystyrene (EPS), Extruded Polystyrene (XPS), Glass Wool (GW), Rock Wool (RW), and Polyurethane Foam (PUR), have been considered as input parameters in the analyses. As for heating sources, considering the study’s emphasis on the less-privileged areas in the rural district where natural gas is unavailable, the investigation has been carried out on four energy sources that are still very much applicable in such areas: Electricity, Fuel Oil, LPG, and Coal. The physical properties and price information of the insulation materials are illustrated in Table 1, while the attributes of the selected fuels are presented in Table 2. The physical properties of the insulation materials listed in Table 1 (thermal conductivity, density, emission factor, and unit price) have been obtained from peer-reviewed literature and technical references rather than from specific commercial products. Accordingly, these values represent widely accepted material characteristics used in building insulation studies and are suitable for comparative analysis. The use of literature-based benchmark values ensures consistency and reproducibility in the optimization process, avoiding variability that may arise from brand-specific commercial products.

**Table 1:** Emission factors, physical properties, and prices of wall components [15,26,27,28,29].

Wall Components	*Emission Factor, f*	*Density, ρ*	*Conductivity, k*	*Price, C*
	(kgCO_2_/kg)	(kg/m^3^)	(W/mK)	(USD/m^3^)
Expanded Polystyrene (EPS)	3.51	20	0.036	100
Extruded Polystyrene (XPS)	3.83	30	0.037	150
Glass Wool (GW)	1.16	22	0.050	75
Rock Wool (RW)	1.47	105	0.040	80
Polyurethane Foam (PUR)	4.47	40	0.036	200
Plaster	0.36	1800	0.87	90
Light Concrete	0.09	1700	0.71	85

**Table 2:** Emission factors, lower heating values, efficiencies, and prices of heating sources [12,30,31].

Heating Source	*Emission Factor, f_h_*	*Lower Heating Value, H_u_*	*Efficiency, η*	*Price, C_f_*
	(kgCO_2_/kWh)		(%)	
Electricity	0.588	3.599 × 10^6^ J/kWh	99	0.1059 USD/kWh
Fuel Oil	0.268	40.594 × 10^6^ J/kg	80	0.734 USD/kg
LPG	0.211	45.980 × 10^6^ J/kg	88	1.921 USD/kg
Coal	0.388	25.080 × 10^6^ J/kg	65	0.273 USD/kg

The evaluation of the ecological effects of thermal insulation is additionally a very significant point determined in this research. Here, the study considers the carbon footprints associated with the manufacture of the insulation material (“embodied” emissions) as well as the ones related to the fuel consumption for space heating (“operational” emissions). As this study concentrates only on the extra insulation that has been placed on the wall, the embodied emission ${(M}_{{\mathrm{CO}}_{2},\mathrm{ins}})$ is determined just for the insulation material. The embodied emissions that are emitted to the atmosphere are represented in Equation (7):

(7) $$M_{{CO}_{2},ins}=\frac{\rho_{ins}x_{ins}f_{ins}}{N}$$

Here, $\rho_{\mathrm{ins}}$ denotes the density of the insulation material, while $f_{\mathrm{ins}}$ represents its emission factor associated with the manufacturing process. The annual CO_2_ emission resulting from fuel consumption during the heating season, $M_{{\mathrm{CO}}_{2},\mathrm{heat}}$, is calculated using Equation (8):

(8) $$M_{{CO}_{2},heat}=\frac{0.024\;HDDf_{h}}{\left(\frac{1}{h_{i}}+\frac{1}{h_{o}}+\frac{x_{plast}}{k_{plast}}+\frac{x_{wall}}{k_{wall}}+\frac{x_{ins}}{k_{ins}}\right)\eta }$$

Consequently, the total amount of carbon dioxide released into the atmosphere as a result of the insulation application and the heating process, $M_{{\mathrm{CO}}_{2},A}$, is defined as the sum of the embodied and operational emissions, as given in Equation (9):

(9) $$M_{{CO}_{2},A}=M_{{CO}_{2},ins}+M_{{CO}_{2},heat}$$

By using this modeling approach, it is possible to choose the best insulation thickness and material economically, as well as in terms of ecological respects.

### 2.4. Multi-Objective Optimization and One-Hot Encoding

Here, a hierarchical optimization strategy is used to achieve the optimal building insulation design. Multi-Objective Flow Direction Algorithm (MOFDA) is used in the first stage to optimize simultaneously the conflicting objectives of cost and CO_2_ emissions, thus generating a Pareto set of non-dominated solutions. The next step involves the selection of the best design as a Multi-Criteria Decision-Making (MCDM) problem. To rank the designs by the Complex Proportional Assessment (COPRAS) method, weighting is performed by both subjective and objective approaches, the latter making use of the entropy method.

#### 2.4.1. Multi-Objective Flow Direction Algorithm (MOFDA)

The Multi-Objective Flow Direction Algorithm (MOFDA) is a meta-heuristic method derived from the Flow Direction Algorithm (FDA) and developed to solve multi-objective optimization problems.

**Figure 2:**
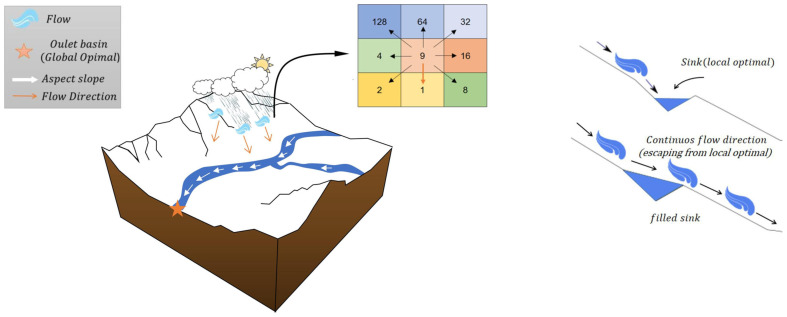
Schematic representation of flow movement toward the basin outlet using the D8 method, and the sink filling process to ensure continuous flow direction [21].

The Flow Direction Algorithm (FDA) is a meta-heuristic optimization method inspired by the process of rainfall turning into surface runoff in a drainage basin. The algorithm is based on the widely used D8 method in hydrology [32]. According to the D8 method, the basin is divided into grid cells, and the flow direction of each cell is determined toward the neighbor with the steepest slope among its eight neighbors [33,34,35]. In FDA, each candidate solution is considered as a “flow,” and the positions and elevations (objective function values) of these flows are defined. The algorithm starts with a randomly generated flow population, and each flow moves toward the position with the lowest elevation (the best objective function value) among its neighboring positions. The velocity of the flow is updated in proportion to the slope between its current point and the target point. Throughout the process, points representing local sinks, which have lower solution quality compared to their neighbors, are filled to prevent the algorithm from becoming stuck in local optima. Consequently, the point where all flows converge and have the highest flow accumulation value is defined as the basin outlet, which is the optimal solution to the problem. Schematic representation of flow movement toward the basin outlet using the D8 method, and the sink filling process to ensure continuous flow direction is given in Figure 2. With this structure, FDA is an effective search algorithm that mimics natural processes and can be adapted to both continuous and discrete optimization problems. For detailed information on the algorithm specifics and its adaptation process to different optimization problems, the study presented by Karami et al. [21] can be examined.

The MOFDA (Multi-Objective Flow Direction Algorithm) adapts the biomimetic process imitation mechanism of FDA into a multi-objective framework [23]. Its primary objective is to determine the Pareto optimal solution set for multiple conflicting objective functions. To achieve this, MOFDA employs three fundamental mechanisms:

- ***Archive Mechanism:*** A fixed-size archive is used to store the best non-dominated Pareto solutions found during the optimization process.
- ***Grid mechanism:*** The area of the objective is split into grids to keep solution diversity when the archive is full. Solutions of the most crowded grids are removed, allowing new solutions to occupy less dense regions. This assures a far-reaching distribution along the Pareto front.
- ***Leader Selection Mechanism:*** The “leader,” which guides other agents in the search space, is selected from the least crowded grid in the archive. Since the archive only contains the best non-dominated solutions, this leader selection mechanism promotes exploration of the least dense areas of the Pareto front.

Through these strategies, MOFDA avoids local optima traps and simultaneously maximizes both solution quality (convergence) and solution distribution (coverage). For detailed information on the algorithm specifics and its adaptation process to different optimization cases, the paper presented by Khodadadi et al. [23] can be examined.

#### 2.4.2. Definition of the Multi-Objective Insulation Optimization Problem

In this study, a multi-objective optimization problem has been formulated to enhance energy efficiency in buildings and minimize environmental impacts. The primary objective of this study is to simultaneously minimize the total cost ($C_{T,A}$) and carbon emissions ($M_{{CO}_{2},A}$) associated with building insulation. Since these two objective functions are conflicting in nature, a Pareto optimal solution set is sought rather than a single optimum point.

The design variables in the optimization problem consist of both categorical and continuous variables. Insulation material (EPS, XPS, GW, RW, and PUR) and heating source (Electricity, Fuel Oil, LPG, and Coal) constitute the categorical variables. In order to incorporate these factors into the numerical optimization algorithm, the one-hot encoding technique was employed. This method involves defining binary variables for each set of categorical features and adding a constraint to ensure that only one variable is active (1) while the others are inactive (0) in each group. As an example, four separate variables are defined for the four different insulation materials, and their sum is limited to 1 ($\sum A_{i}=1$). The insulation thickness (Y_t_) is included in the model as a continuous variable with values from 0.01 m to 0.4 m.

Accordingly, the design variable vector is defined as $x=\left[A_{EPS},A_{XPS},A_{GW},A_{RW},A_{PUR},B_{Elec},B_{Fuel\;oil},B_{LPG},B_{Coal},Y_{t}\right]$, and the mathematical model of the problem is presented in Equation (10):

(10) s.t. minCTx,MCO2x∑i=15Ai=1 , Ai ∈0,1 Insulation materials, One−hot encoding∑j=14Bj=1 , Bj ∈0,1 (Heating sources, One−hot encoding)0.01 <Yt<0.40 (Insulation thickness)

where

- A_i_: Insulation materials represented by one-hot encoding (EPS, XPS, GW, RW, PUR),
- B_j_: Heating sources represented by one-hot encoding (Electricity, Fuel Oil, LPG, Coal)
- Y_t_: Insulation thickness in meters.

#### 2.4.3. COPRAS Method and Shannon Entropy-Based Weighting

Multi-criteria decision-making (MCDM) methods are typically used in various engineering studies to decide the best option from a set of alternatives [36,37,38]. Here, the COPRAS (Complex Proportional Assessment) technique was applied to figure out the insulation parameters leading to the most Pareto front from MOFDA. The COPRAS method is the most popular one in multi-criteria decision-making problems because of its ease of use [39]. This method, created by Zavadskas et al. [40], performs the ranking of the alternatives by proportionally assessing their beneficial and non-beneficial features. The mathematical representation of the method is given below:

***Step 1: The initial decision-making matrix (X).***

The initial decision-making matrix (X) with m alternatives and n criteria is created.

(11) $${X=[x_{ij}]}_{mn}=\left[\begin{array}{cccc} x_{11} & x_{12} & \ldots & x_{1n}\\ x_{21} & x_{22} & \ldots & x_{2n}\\ \vdots & \vdots & \vdots & \vdots \\ x_{m1} & x_{m2} & \ldots & x_{mn} \end{array}\right]$$

***Step 2: Normalization (R)*.**

To make the criteria with different units comparable, the matrix is normalized:

(12) $$R={\left[r_{ij}\right]}_{mn}=\;\frac{x_{ij}}{\sum_{i=1}^{m}x_{ij}}$$

**Step 3: Calculating the Weighted Matrix (D).**

Creation of a weighted matrix with the weight coefficient determined for each criterion:

(13) $$D={\left[d_{ij}\right]}_{mn}=\;r_{ij}\;\cdot \;w_{j}$$

**Step 4: Calculating the Sums of Beneficial and Non-Beneficial Criteria.**

For each alternative i, the sums of the weighted normalized values for the beneficial criteria $S_{+i}$ and the non-beneficial (negative) criteria $S_{-i}$ are calculated.

(14) $$S_{+i}=\sum_{İ=1}^{m}d_{+ij}$$

(15) $$S_{-i}=\sum_{İ=1}^{m}d_{-ij}$$

**Step 5: Calculating the Relative Importance Value (** $Q_{i}$ **).**

The $Q_{i}$ value, which indicates the final success of each alternative, is calculated using the following formula:

(16) $$Q_{i}=\;S_{+i}+\frac{\sum_{i=1}^{m}S_{-i}}{S_{-i}\cdot \sum_{i=1}^{m}\left(\frac{1}{S_{-i}}\right)}$$

**Step 6: Performance Degree and Ranking.**

The alternative with the highest value is considered the best solution. The benefit rating of other alternatives is calculated as a percentage according to the best solutions, as follows:

(17) $$U_{i}=\;\frac{Q_{i}}{Q_{max}}\cdot \%100$$

#### 2.4.4. Determination of Criterion Weights

The optimal solution choice from the resulting Pareto set is a direct function of the criterion importance weights (w_j_). The weighting factor is an essential stage that determines the final choice directly. To obtain human-intervention-free results, objective methods like the entropy method are usually chosen. We have created two different scenarios in this research to compare the influence of the weight assignment method on the outcome: the subjective assignment and the entropy method.

***Subjective Weighting***

Here, weights are explicitly established by the researcher, who considers the trade-off between cost and environmental impact.

***Entropy-Based Objective Weighting***

One of the preferred approaches for objective weighting in engineering studies is the Shannon Entropy method. Using the Shannon Entropy method [41], which measures the uncertainty and amount of information in a dataset, the weights were calculated entirely based on the data distribution obtained from the MOFDA results. The application steps of the entropy method are as follows:

**Step 1:** Development of the initial decision matrix (X).

(18) $${X=[x_{ij}]}_{mn}=\left[\begin{array}{cccc} x_{11} & x_{12} & \ldots & x_{1n}\\ x_{21} & x_{22} & \ldots & x_{2n}\\ \vdots & \vdots & \vdots & \vdots \\ x_{m1} & x_{m2} & \ldots & x_{mn} \end{array}\right]$$

**Step 2:** Normalization of the decision matrix. Each value is normalized as $p_{ij}$ using Equation (19).

(19) $$p_{ij}=\frac{x_{ij}}{\sum_{İ=1}^{m}x_{ij}},\;\forall i,j\;$$

**Step 3:** Calculation of the entropy $E_{j}$ for the j-th indicator.

(20) $$E_{j}=-{\left(\ln m\right)}^{-1}\sum_{i=1}^{m}p_{ij}\ln p_{ij},\;\forall j$$

**Step 4:** Calculation of the distance (divergence) $d_{j}$.

(21) $$d_{j}=1-E_{j}$$

**Step 5:** Determination of the criterion weights $w_{j}$.

(22) $$w_{j}=\frac{d_{j}}{\sum_{j=1}^{n}d_{j}},\;\forall j\;$$

## 3. Results and Discussion

**Figure 3:**
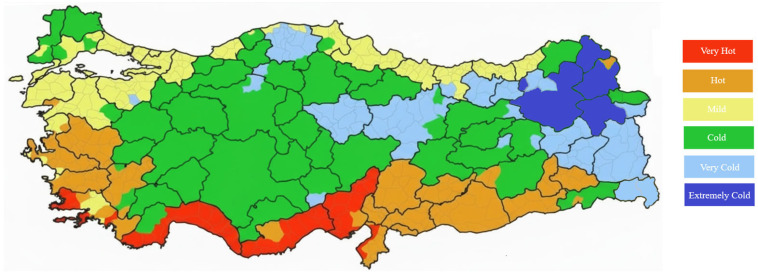
Updated climatic zones of Türkiye based on the new six-region classification.

With the recent updates introduced in the TS 825 standard, which forms the basis for evaluating the energy performance of buildings in Türkiye, a more refined climatic classification approach has been adopted. While previous studies and the former version of the standard defined four degree-day regions nationwide, the new classification system divides Türkiye into six distinct climatic zones ranging from “Very Hot” to “Extremely Cold.” Figure 3 illustrates the updated climatic zone map developed according to this new framework. This enhanced approach enables a more precise assessment of region-specific energy demands.

In this study, pilot provinces with distinct climatic characteristics are selected in order to clearly observe the influence of climatic diversity on insulation optimization and carbon emissions. The map displays the locations of the provinces as well as the distribution of their Heating Degree Day (HDD) values. The range of the HDD values of the selected provinces is from 359 to 4150, which depicts the extended energy demand profile of Turkey that varies from the mild coastal climate to the harsh continental climate of the eastern regions.

**Figure 4:**
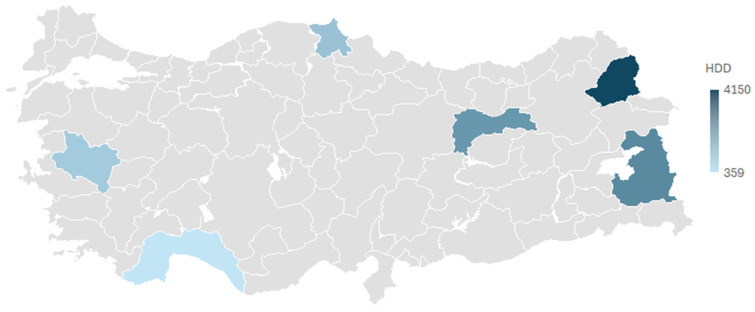
Selected cities and the geographical distribution of Heating Degree Days (HDD).

In earlier sections, it was explained that the annual average HDD values used in the analyses are derived from the consideration of the effect of solar radiation. The distribution shown in Figure 4 reflects that Heating Degree Days (HDD) values, which are directly proportional to heating energy demand, change significantly in different locations. In particular, the areas that are shown in dark color with higher HDD values (e.g., the eastern provinces on the map) point to a heating demand that is many times higher per unit area than that of the lighter-colored coastal regions. Consequently, the results of the economic and environmental assessments for these areas will likely differ dramatically in terms of the optimal insulation thickness and the choice of fuel.

The study uses a multi-objective optimization framework based on the Multi-Objective Flow Direction Algorithm (MOFDA) to find the optimal values of the building envelope design parameters. The main objective of the study is to reduce the life-cycle cost and the environmental impact represented by CO_2_ emissions at the same time. As a result of the multi-objective optimization problem, which is naturally of this type, there is no single solution that meets both conflicting objectives. Thus, the set of solutions that represent different trade-offs between cost and emissions is generated, i.e., the Pareto front. It allows researchers to choose the design alternatives that meet their budget and environmental priorities.

The Complex Proportional Assessment (COPRAS) method is used as a multi-criteria decision-making method to select the most appropriate design options from the Pareto solution set. A hybrid weighting strategy is used to determine the weighting factors involved in the decision-making process:

- **Subjective Weighting:** Three cost–emission preference scenarios specified by the researcher (75% Cost–25% Emissions, 50% Cost–50% Emissions, 25% Cost–75% Emissions).
- **Objective Weighting:** The Shannon Entropy method, which evaluates the variability and information content within the dataset.
- **Single-Objective Cases:** Extreme scenarios where either cost or emissions are assigned a full weight of 100%.

**Figure 5:**
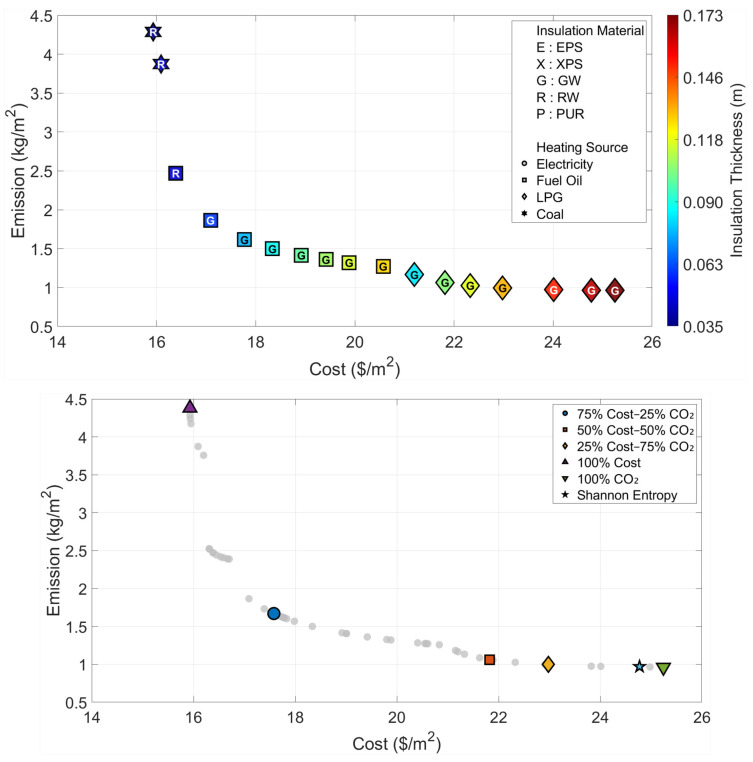
Optimal insulation solutions identified by different objective weighting and decision-making criteria (Region I).

**Figure 6:**
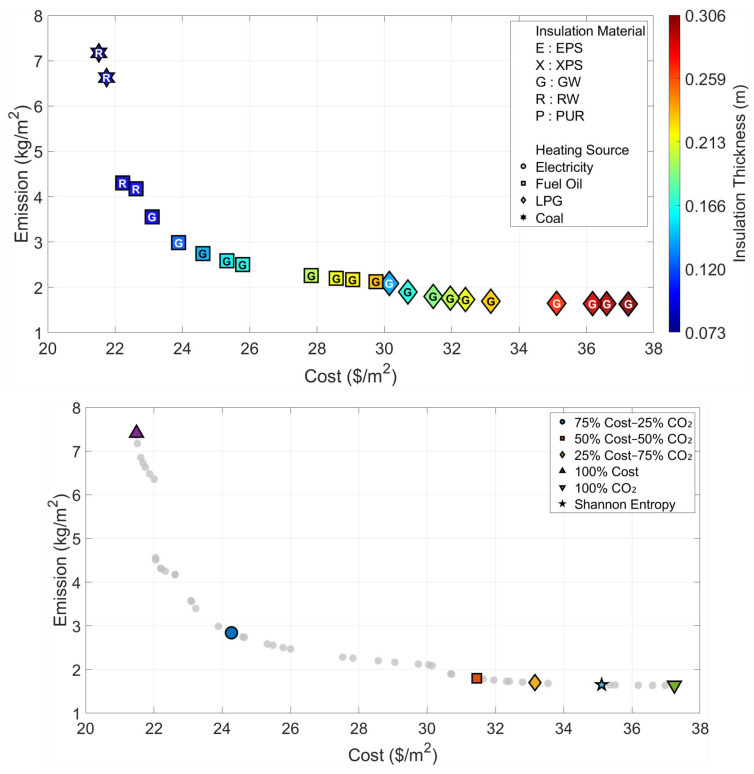
Optimal insulation solutions identified by different objective weighting and decision-making criteria (Region II).

**Figure 7:**
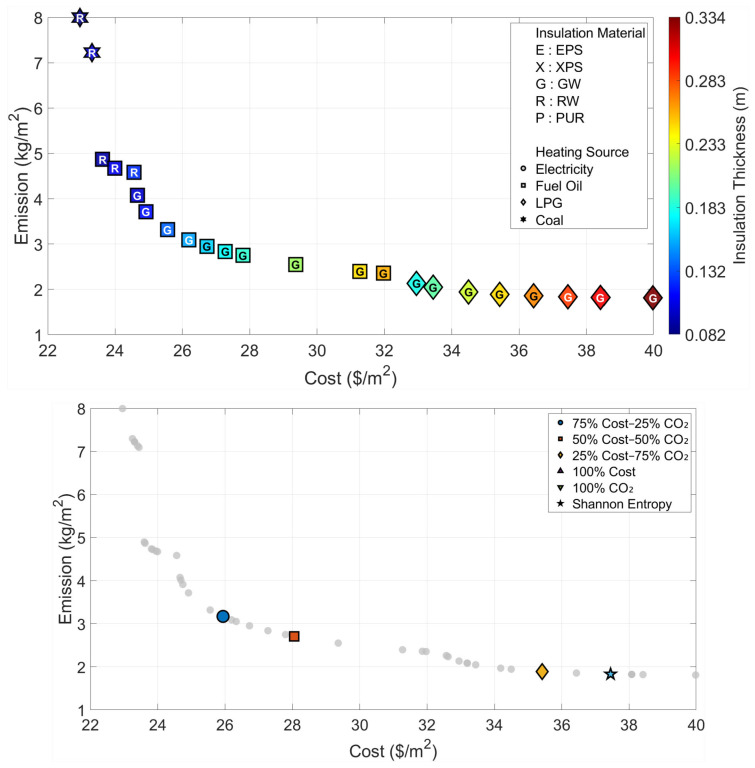
Optimal insulation solutions identified by different objective weighting and decision-making criteria (Region III).

**Figure 8:**
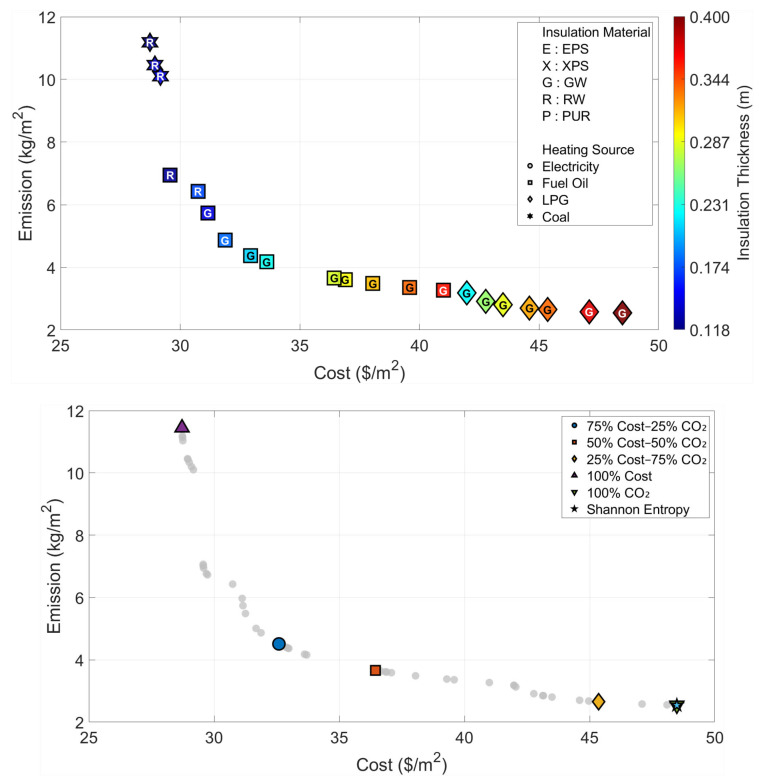
Optimal insulation solutions identified by different objective weighting and decision-making criteria (Region IV).

**Figure 9:**
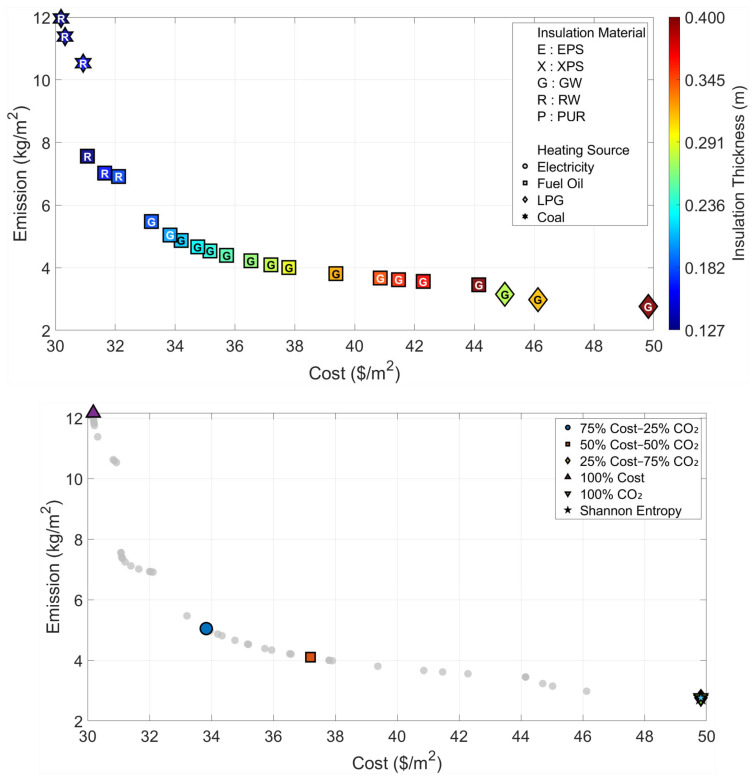
Optimal insulation solutions identified by different objective weighting and decision-making criteria (Region V).

**Figure 10:**
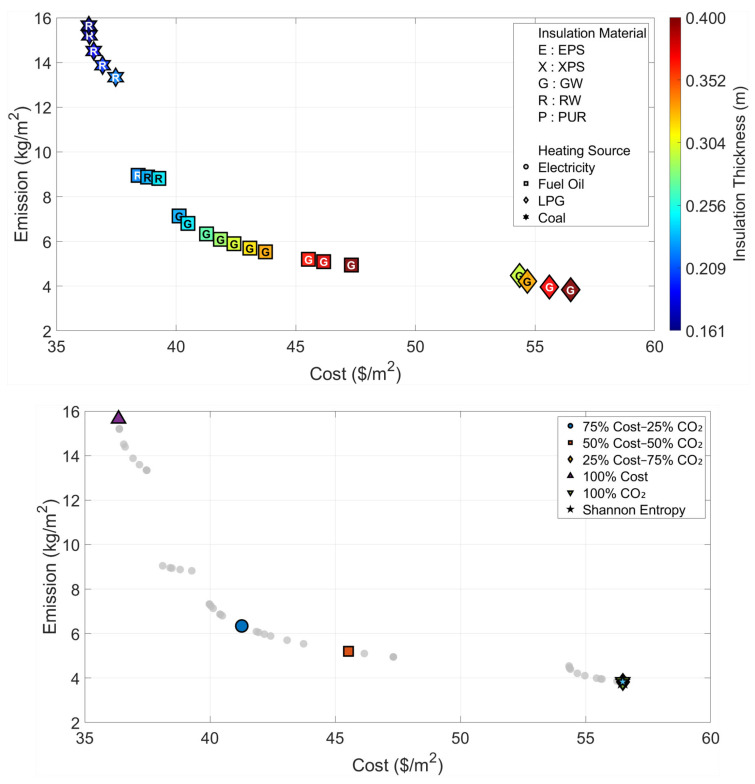
Optimal insulation solutions identified by different objective weighting and decision-making criteria (Region VI).

All optimization and decision-making operations are carried out independently for the six climatic zones that are considered in the paper. The Pareto fronts and the solution spaces of the MOFDA for each area are shown in Figure 5, Figure 6, Figure 7, Figure 8, Figure 9 and Figure 10. Interpretability is improved, and analytical depth is demonstrated by the results that are visualized in a two-part graphical layout. The top figure displays the entire solution space. In these figures, the color scale denotes the insulation thickness, the characters indicate the insulation material type (E: EPS, X: XPS, G: GW, R: RW, and P: PUR), and different symbols show the type of heating source used. The bottom figure shows only the “selected” optimal points for the six weighting scenarios found by the COPRAS and Shannon Entropy methods; thus, visual complexity is avoided.

The optimal design variables for each climatic zone and six weighting scenarios, such as insulation material, insulation thickness, and heating source, are given in Table 3, Table 4, Table 5, Table 6, Table 7 and Table 8. The tables exhibit how building design decisions differ based on the set of priorities, whether they are cost-oriented or environmentally oriented.

The multi-objective optimization analysis for the warmest climatic region (Region I) showed a strong inverse correlation between the cost and emission objectives, as demonstrated by the Pareto front in Figure 5. The system uses Coal as the most economical heating source and chooses Rock Wool insulation with a thickness of 3.3 cm when the optimum point for minimum cost (Optimum Cost) is looked at, thus the unit cost comes to 15.93 USD/m^2^. On the other hand, taking the optimum point corresponding to minimum emissions (Optimum CO_2_), the insulation thickness is increased almost five times to 17.3 cm, the insulation material is changed to Glass Wool, and the heating source is changed to LPG, which is a cleaner fuel option.

Furthermore, an evaluation of the decision-making scenarios presented in Table 3 shows that the Shannon Entropy method assigns a weight of 92% to emissions due to the distribution characteristics of the dataset. Consequently, it recommends a solution that is highly similar to the “Optimum CO_2_” scenario, consisting of 16.5 cm of Glass Wool insulation combined with LPG as the heating source.

**Table 3:** Multi-objective optimization results under different Cost–CO_2_ weighting scenarios (Region I).

	Cost	CO_2_	Cost	CO_2_	x	Insulation	Heating
Weighting	(%)	(%)	($/m^2^)	(kg/m^2^)	(m)	Material	Source
	75	25	17.58	1.67	0.072	Glass Wool	Fuel Oil
	50	50	21.82	1.06	0.105	Glass Wool	LPG
	25	75	22.98	1.00	0.132	Glass Wool	LPG
Optimum Cost	100	0	15.93	4.38	0.033	Rock Wool	Coal
Optimum CO_2_	0	100	25.24	0.96	0.173	Glass Wool	LPG
Shannon Entropy	8	92	24.77	0.97	0.165	Glass Wool	LPG

The results obtained for Region II, which represents a transition from a temperate climate to cooler conditions (Figure 6 and Table 4), indicate a distinct increase in optimal insulation thicknesses parallel to the rise in heating load compared to Region I. In the cost-oriented approach, the use of 6.9 cm-thick Rock Wool and Coal is deemed sufficient with a cost of 21.49 USD/m^2^; however, in the “Optimum CO_2_” scenario, where environmental sensitivity is prioritized, the insulation thickness reaches 30.6 cm, and the material preference is Glass Wool and LPG as heating source. In this region, where the Shannon Entropy method assigns an 89% weight to emissions, the decision mechanism recommends the solution with Glass Wool of 26.8 cm thickness and LPG fuel, featuring a cost of 35.11 USD/m^2^ and an emission value of 1.65 kg/m^2^, as the most balanced option.

**Table 4:** Multi-objective optimization results under different Cost–CO_2_ weighting scenarios (Region II).

	Cost	CO_2_	Cost	CO_2_	x	Insulation	Heating
Weighting	(%)	(%)	($/m^2^)	(kg/m^2^)	(m)	Material	Source
	75	25	24.27	2.84	0.133	Glass Wool	Fuel Oil
	50	50	31.45	1.80	0.190	Glass Wool	LPG
	25	75	33.16	1.70	0.230	Glass Wool	LPG
Optimum Cost	100	0	21.49	7.41	0.069	Rock Wool	Coal
Optimum CO_2_	0	100	37.24	1.64	0.306	Glass Wool	LPG
Shannon Entropy	11	89	35.11	1.65	0.268	Glass Wool	LPG

When Region III is evaluated in light of the Pareto front in Figure 7 and the data in Table 5, it is observed that the gap between cost and emission objectives widens as the climate becomes harsher. While the use of 8.1 cm-thick Rock Wool (22.95 USD/m^2^) emerges as the optimum solution when only economic parameters are considered, this thickness increases approximately fourfold to reach 33.9 cm in a design aiming to minimize the carbon footprint. Specifically for this region, the design parameters selected as a result of the weights determined by the Shannon Entropy method (28.8 cm Glass Wool and LPG) draw a profile very close to the 100% CO_2_-oriented extreme scenario; this situation indicates that the data distribution renders environmental criteria more dominant.

**Table 5:** Multi-objective optimization results under different Cost–CO_2_ weighting scenarios (Region III).

	Cost	CO_2_	Cost	CO_2_	x	Insulation	Heating
Weighting	(%)	(%)	($/m^2^)	(kg/m^2^)	(m)	Material	Source
	75	25	25.14	3.17	0.147	Glass Wool	Fuel Oil
	50	50	28.05	2.71	0.191	Glass Wool	Fuel Oil
	25	75	35.42	1.89	0.248	Glass Wool	LPG
Optimum Cost	100	0	22.95	8.03	0.081	Rock Wool	Coal
Optimum CO_2_	0	100	40.33	1.81	0.339	Glass Wool	LPG
Shannon Entropy	12	88	37.45	1.83	0.288	Glass Wool	LPG

In the analyses of Region IV, where the effects of the cold climate zone are felt (Figure 8 and Table 6), it is noteworthy that the increase in energy demand pushes the optimization results to the limits. Even the cost optimization scenario suggests a significant insulation thickness of 11.3 cm (Rock Wool), whereas in the environmental optimum scenario, the insulation thickness reaches the upper limit of 40 cm determined in the model. In this region, where the Shannon Entropy method assigns an 89% emission weight, the objective decision-making mechanism directly overlaps with the “Optimum CO_2_” point, indicating the use of Glass Wool and LPG at the maximum thickness (40 cm) as the most suitable solution. This situation reveals that radical insulation measures are inevitable to ensure environmental sustainability in cold regions.

**Table 6:** Multi-objective optimization results under different Cost–CO_2_ weighting scenarios (Region IV).

	Cost	CO_2_	Cost	CO_2_	x	Insulation	Heating
Weighting	(%)	(%)	($/m^2^)	(kg/m^2^)	(m)	Material	Source
	75	25	32.59	4.51	0.202	Glass Wool	Fuel Oil
	50	50	36.44	3.66	0.283	Glass Wool	Fuel Oil
	25	75	45.35	2.65	0.336	Glass Wool	LPG
Optimum Cost	100	0	28.72	11.45	0.113	Rock Wool	Coal
Optimum CO_2_	0	100	48.48	2.54	0.400	Glass Wool	LPG
Shannon Entropy	11	89	48.48	2.54	0.400	Glass Wool	LPG

The findings for Region V, which exhibits climate characteristics similar to Region IV but has a higher number of heating degree days, are presented in Figure 9 and Table 7. In the cost-oriented approach, while the unit cost rises to the level of 30.18 USD/m^2^, the system selects the combination of 12.4 cm-thick Rock Wool and Coal. In contrast, in the scenario where emissions are minimized, the maximum insulation thickness limit of 40 cm is reached, just as in the previous region, and LPG fuel is preferred. The fact that the Shannon Entropy approach assigns a very high weight of 90% to emissions confirms that carbon emission, rather than cost, is the determining factor in the decision process in this region, and directly points to the most environmentally friendly solution (identical to the Optimum CO_2_ scenario).

**Table 7:** Multi-objective optimization results under different Cost–CO_2_ weighting scenarios (Region V).

	Cost	CO_2_	Cost	CO_2_	x	Insulation	Heating
Weighting	(%)	(%)	($/m^2^)	(kg/m^2^)	(m)	Material	Source
	75	25	33.83	5.05	0.203	Glass Wool	Fuel Oil
	50	50	37.20	4.10	0.281	Glass Wool	Fuel Oil
	25	75	49.81	2.76	0.400	Glass Wool	LPG
Optimum Cost	100	0	30.18	12.17	0.124	Rock Wool	Coal
Optimum CO_2_	0	100	49.81	2.76	0.400	Glass Wool	LPG
Shannon Entropy	10	90	49.81	2.76	0.400	Glass Wool	LPG

The analyses pertaining to Region VI, which possesses the harshest climate conditions in Türkiye (Figure 10 and Table 8), demonstrate the critical role of the building envelope design under extreme weather conditions. In this region, even when the most economical solution (Optimum Cost) is sought, the system suggests a remarkably high insulation thickness of 16.1 cm; this situation proves that insulation is a necessity not only environmentally but also economically in harsh climates. In the scenario where the environmental impact is minimized, although the cost rises to the level of 56.48 USD/m^2^, the use of Glass Wool and LPG at the maximum thickness of 40 cm is required to keep emissions at the level of 3.83 kg/m^2^. The fact that the Shannon Entropy method points to the environmental optimum point for this region as well indicates that sustainable design in extremely cold climates is only possible with high-performance insulation strategies.

**Table 8:** Multi-objective optimization results under different Cost–CO_2_ weighting scenarios (Region VI).

	Cost	CO_2_	Cost	CO_2_	x	Insulation	Heating
Weighting	(%)	(%)	($/m^2^)	(kg/m^2^)	(m)	Material	Source
	75	25	41.27	6.34	0.271	Glass Wool	Fuel Oil
	50	50	45.53	5.20	0.367	Glass Wool	Fuel Oil
	25	75	56.48	3.83	0.400	Glass Wool	LPG
Optimum Cost	100	0	36.35	15.65	0.161	Rock Wool	Coal
Optimum CO_2_	0	100	56.48	3.83	0.400	Glass Wool	LPG
Shannon Entropy	10	90	56.48	3.83	0.400	Glass Wool	LPG

Just as shown in Table 3, Table 4, Table 5, Table 6, Table 7 and Table 8, the optimum insulation thicknesses obtained are in line with those reported by other studies in the literature as well. As an example, Aktemur and Atikol [42] investigated optimal insulation thicknesses throughout the climatic regions of Türkiye and henceforth reported values that varied from almost 2.8 cm to 45.1 cm based on the insulation material, wall structure, and fuel type. This wide range not only covers the low economic optimum values but also the high insulation requirements found in the coldest regions in the current study. Similarly, Uçar [43] mentioned typical optimum thicknesses that lie between 2.5 cm and 13 cm for residential units when the cost- and emission-based criteria are taken into account, which is very much in accordance with our results for the mild and temperate climatic zones. Moreover, Timuralp [44] and Kültürel and Dağkurs [45] revealed that the optimum insulation thickness could be raised up to 20–25 cm if different types of walls, environmental priorities, and post-disaster reconstruction aspects are considered. Consequently, the optimal insulation thicknesses that have been figured out in this paper, particularly those related to both the economic and the CO_2_-oriented scenarios, lie within the reported ranges in the literature and hence verify the validity and relevance of the findings for various climatic situations.

To highlight our data agreement with the previous works even more, and to demonstrate the methodological novelty and contribution of the current paper, Table 9 summarizes and compares the main characteristics and research dimensions of recent literature.

## 4. Conclusions

In this study, a multi-objective optimization framework aimed at the simultaneous minimization of life-cycle costs and CO_2_ emissions in external wall insulation design is developed using the Multi-Objective Flow Direction Algorithm (MOFDA) integrated with a One-Hot Encoding scheme. The analyses conducted for Türkiye’s updated six climatic regions demonstrate the decisive influence of climatic conditions on the optimal design parameters.

According to the results, the cost-oriented optimum insulation thickness in Region I, which represents the warmest climate, is 3.3 cm with Rock Wool, whereas the emission-minimizing optimum thickness increases to 17.3 cm with Glass Wool. When climatic severity changes dramatically across different areas, the optimum thickness also shows substantial variations. As an example, the cost optimum is 6.9 cm in Region II, while it increases to 16.1 cm in Region VI, which is the coldest region. The 40 cm insulation thickness identified in the coldest regions represents the upper limit imposed in the optimization model. In theory, achieving even lower CO_2_ emissions would require insulation thicknesses greater than 40 cm; however, such values fall outside practical feasibility. In real-world applications, insulation layers often range between approximately 3 and 20 cm due to architectural, structural, and installation constraints. Therefore, while the model demonstrates that extreme insulation measures are required to approach minimum-emission scenarios in very cold climates, it also highlights that current construction practices and material technologies limit the extent to which insulation alone can reduce CO_2_ emissions. This indicates that the theoretical optimum lies beyond what is practically achievable, reinforcing the notion that sustainability in such climates may necessitate complementary measures or alternative solutions in addition to insulation thickness.

The multi-criteria decision-making analyses highlight a clear typical change in the selection of fuel, depending on the different weighting scenarios. When balanced weighting conditions are applied, meaning that cost and CO_2_ emissions are taken into account equally (50–50%), LPG is the source of heating that turns out to be the most advantageous in the milder climates of Regions I and II, while Fuel Oil becomes the best energy source in Regions III, IV, V, and VI, where the heating demand is significantly higher. The transition here is a demonstration of how the increase in climatic severity changes the trade-off between unit energy cost and emission factors.

In the case of objective weighting by the Shannon Entropy method, carbon dioxide emissions are always the main factor influencing the situation compared to costs, with weights given between 88% and 92% in all areas. The reason for this is mainly the proportional variation characteristics of the Pareto front. While the cost values are within a relatively narrow range with a slight variation (approximately twofold), the emission values depict a much steeper and wider proportional change (approximately eightfold). The Shannon Entropy method considers such a high variability and distinctiveness as a high information content, and thus, it assigns more importance to the emission criterion.

In general, using One-Hot Encoding with MOFDA has shown to be a very powerful method to deal with complex building optimization problems that have discrete variables (insulation material, fuel type) and continuous variables (insulation thickness). The findings highlight that, rather than relying on a uniform solution, the development of climate-specific, practically feasible, and priority-driven (cost versus environmental impact) insulation strategies is essential for achieving optimal building performance.

In addition to showing the ability of the MOFDA One-Hot Encoding framework to find cost- and CO_2_-efficient insulation setups for six climate zones, this research leaves room for future work. Subsequent research may consider more design variables like different types of wall materials, window-to-wall ratios, renewable energy use, or hybrid heating systems to expand the optimization scope. In addition, future research may consider different multi-criteria decision-making methods and weighting techniques for comparison with the COPRAS-Entropy pair used in this paper. It would also be good to use stochastic or probabilistic methods to analyze uncertainties in fuel prices, emission factors, service life, and thermal properties to increase the robustness and practical reliability of the work. Most importantly, researchers may use actual energy data from buildings that have been monitored or run experiments in a controlled environment to validate the optimization-based results better and help real-world applications.

## Figures and Tables

**Table 9:** Comparative analysis between the present study and recent literature.

Study	Approach	Decision Variables	Objective Functions	Climate Zones	Decision Making Method
Behzadi Hamooleh et al. [46]	RSM	Temperature of thermostat, Insulation thickness, Insulation material	Thermal Comfort, Energy consumption	Four climate zones	None
Wang et al. [46]	NSGA-II	U-values, Window-to-wall ratios (N, S, E, W)	Energy Consumption, Thermal Discomfort	Single climate zone	Ideal Point Method
Wang et al. [47]	Analytical Method	Insulation thickness, Insulation material, Fuel type	Economic cost, Energy consumption, Carbon emissions	Single climate zone	Balanced Index Method
Uçar [43]	Analytical	Material, Cost parameters	Economic cost, Carbon emissions	Single zone	None
Timuralp et al. [44]	Simulation	Wall type, Material	Economic cost	Three climate zones	None
This study	MOFDA + One-Hot Encoding	Insulation thickness, Insulation material, Fuel type, Climate zone	Economic cost, Carbon emissions	Six climate zones	COPRAS + Shannon Entropy

## Data Availability

The original contributions presented in the study are included in the article, further inquiries can be directed to the corresponding author.
